# Supplementary material for: Inhalable biohybrid microrobots: a non-invasive approach for lung treatment
Source: Nat Commun. 2025 Jan 14;16:666. doi: 10.1038/s41467-025-56032-4 (PMC11733022; doi:10.1038/s41467-025-56032-4)
Supplement: Supplementary file 1 — Supplementary Information [file 41467_2025_56032_MOESM1_ESM.pdf]

Supplementary Information

Inhalable biohybrid microrobots: a non-invasive approach for lung treatment

Zhengxing Li[1,2†], Zhongyuan Guo[1†], Fangyu Zhang[1†], Lei Sun[1†], Hao Luan[1,2], Zheng Fang[1], Jeramy L. Dedrick[3], Yichen Zhang[2], Christine Tang[1], Audrey Zhu[1], Yiyan Yu[1], Shichao Ding[1], Dan Wang[1], An-Yi Chang[1], Lu Yin[1], Lynn M. Russell[3], Weiwei Gao[1], Ronnie H. Fang[1,4], Liangfang Zhang[1,2]* and Joseph Wang[1,2]*

1. Aiiso Yufeng Li Family Department of Chemical and Nano Engineering, University of California San Diego, La Jolla, CA 92093, United States.

2. Program in Materials Science and Engineering, University of California San Diego, La Jolla, CA 92093, United States.

3. Scripps Institution of Oceanography, University of California San Diego, La Jolla, CA 92093, United States.

4. Division of Host-Microbe Systems and Therapeutics, Department of Pediatrics, University of California San Diego, La Jolla, CA 92093, United States.

† These authors contributed equally to this work.

* Correspondence should be addressed to J.W. (josephwang@ucsd.edu) and L.Z. (zhang@ucsd.edu)

Table of contents:

Supplementary Figures 1 to 33.

Supplementary Table 1.

Supplementary Video legends 1 to 8.

Supplementary Figures

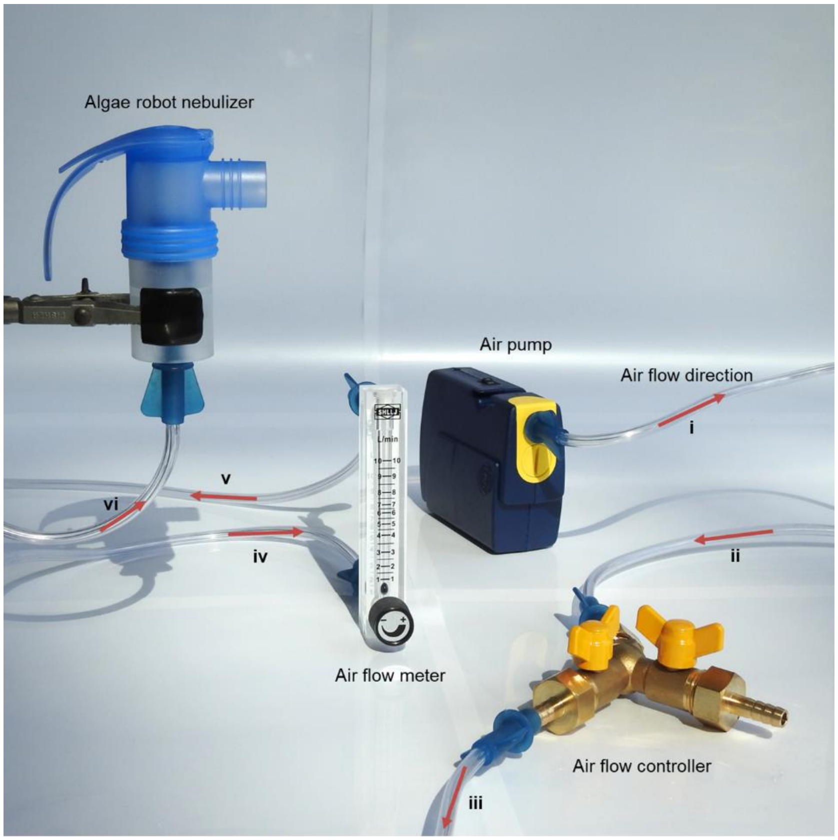

Supplementary Fig. 1. Algae robot nebulizer system. The air pump generates a constant air flow, the air flow controller and air flow meter adjust the system's air flow rate, and the algae robot nebulizer generates the aerosol flow based on the system's air flow, from steps i to vi.

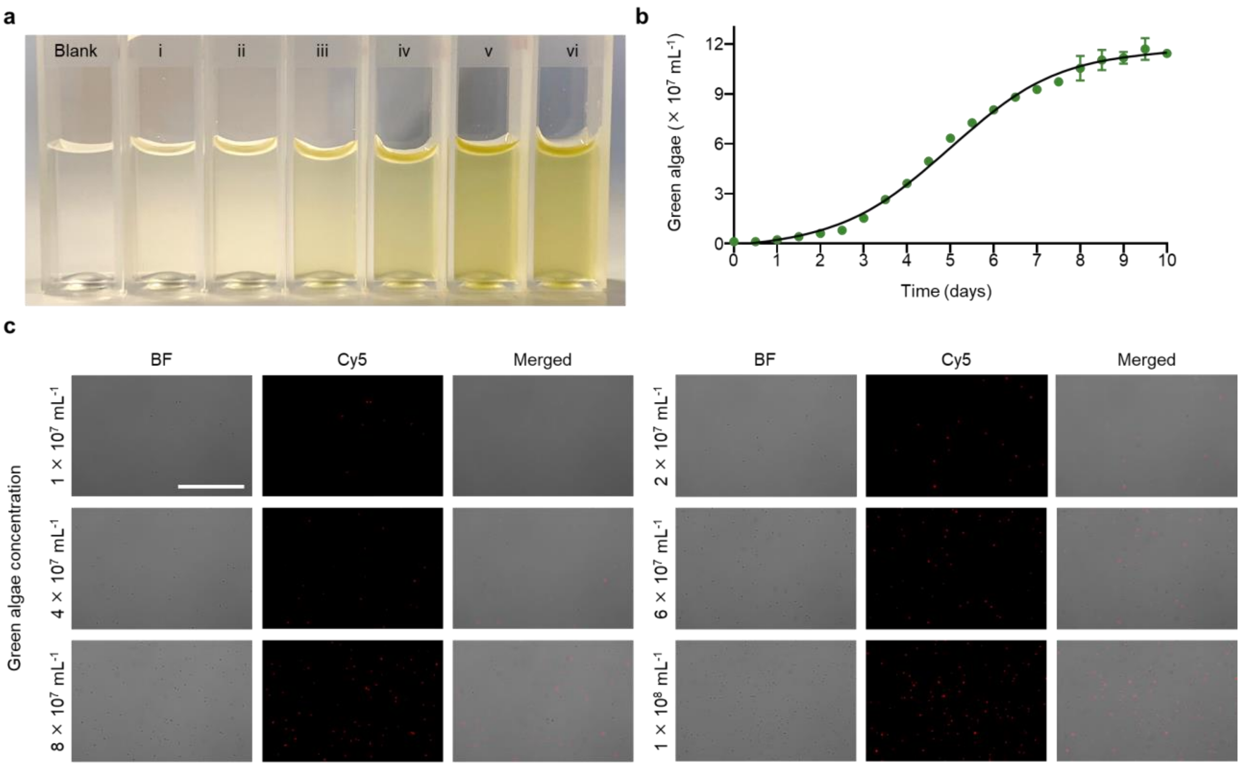

Supplementary Fig. 2. Green algae, *M. pusilla*, cultured in L1-Si medium at room temperature (22 °C). a, Photograph of solutions containing different green algae concentrations. Blank, pure L1-Si medium; i, 1×10^7 mL^{-1}; ii, 2×10^7 mL^{-1}; iii, 4×10^7 mL^{-1}; iv, 6×10^7 mL^{-1}; v, 8×10^7 mL^{-1}; vi, 1×10^8 mL^{-1}; **b,** Green algae growth curve over 10 days in L1-Si medium at 22 °C (n = 3, mean ± s.d.). **c,** Representative brightfield (BF), fluorescence (Cy5), and merged microscopy images of green algae at different concentrations. Scale bar, 100 µm.

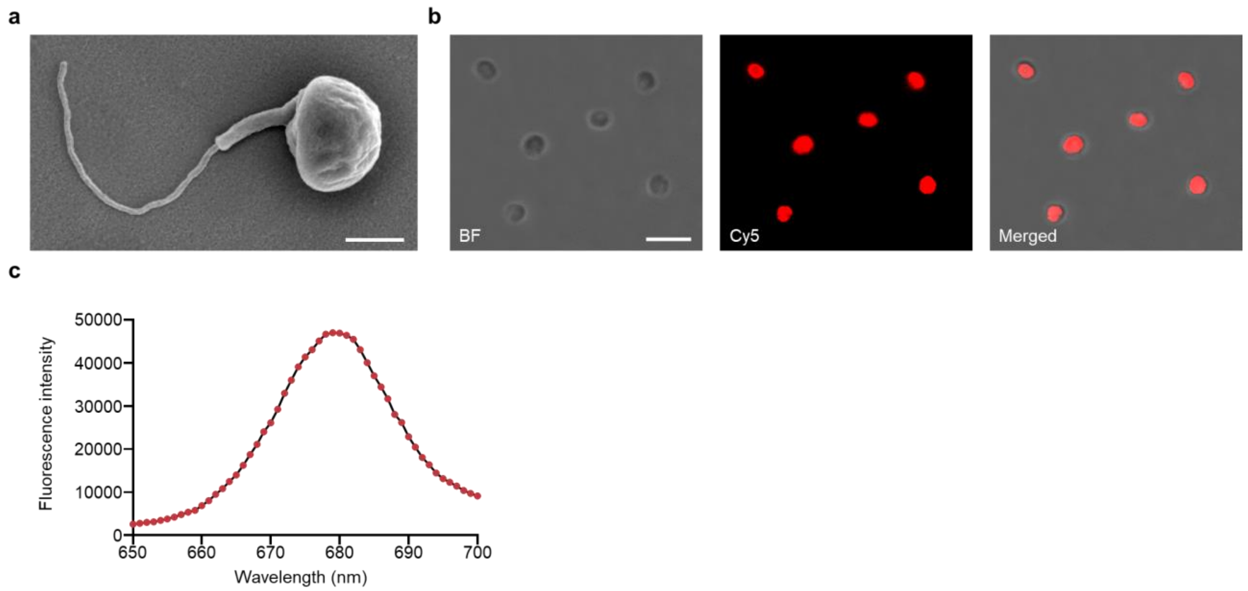

Supplementary Fig. 3. Characterization of algae robot morphology. a, Representative SEM image of an algae robot. Scale bar, 500 nm. **b,** Representative confocal fluorescence images of algae robots. Scale bar, 2 µm. **c,** The optical spectra of algae robots in L1-Si culture medium at 22 °C.

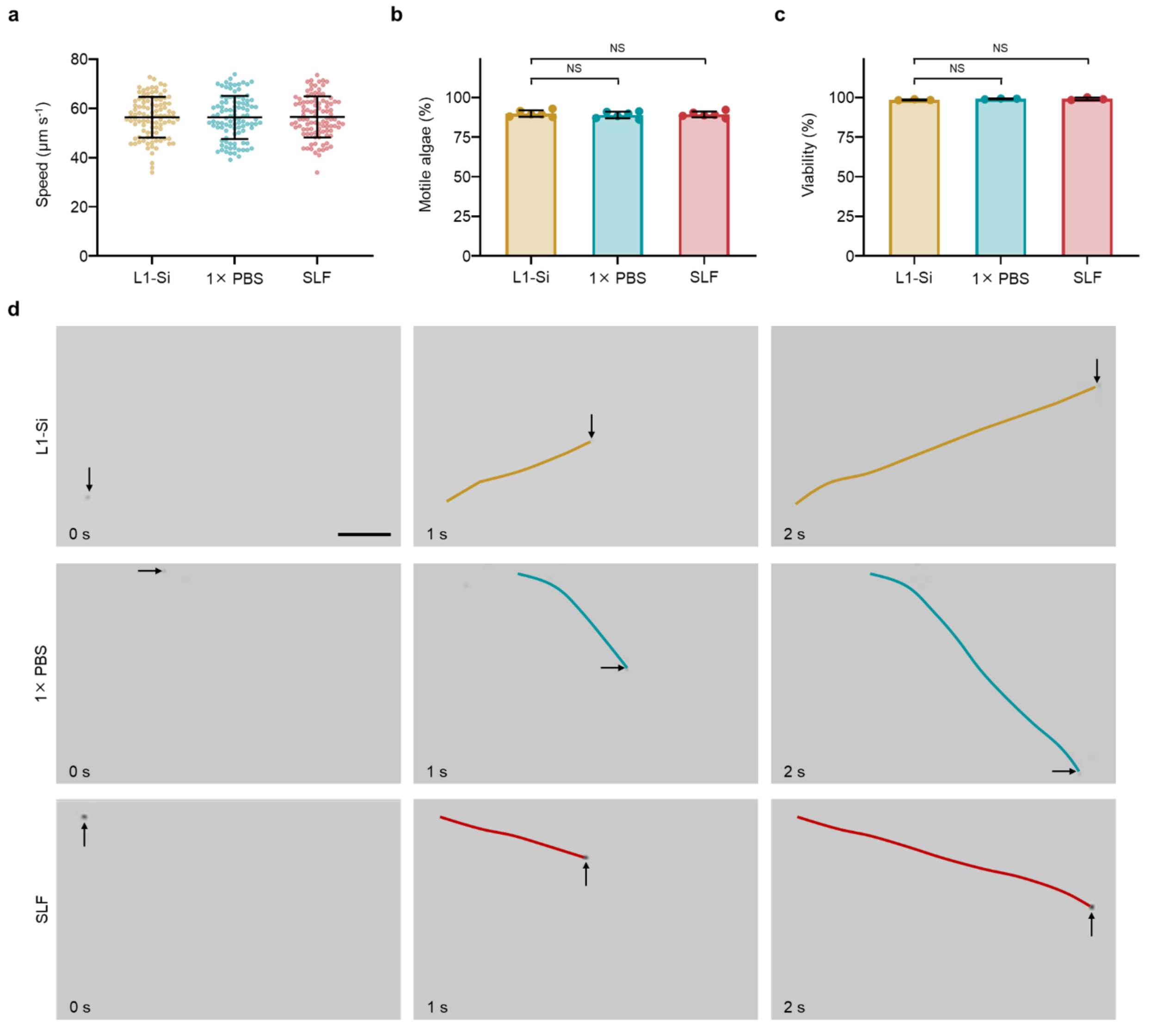

Supplementary Fig. 4. Motility of algae robots in different media at room temperature (22 °C). **a,** Speed of algae robot in L1-Si culture medium, 1× PBS buffer, and simulated lung fluid (SLF) (n = 100, mean ± s.d.). **b,** Corresponding motility ratio of algae robots in three different media (n = 6, mean ± s.d.). **c,** Relevant viability of algae robot in three different media (n = 3, mean ± s.d.). **d,** Representative 0, 1, and 2-s trajectories of a single algae robot in three different media. The black arrow points to the algae robot. Scale bar, 20 μm. Statistical analysis for the motility ratio and viability of algae robot were performed using repeated-measure one-way analysis of variance (ANOVA). NS: $P > 0.05$.

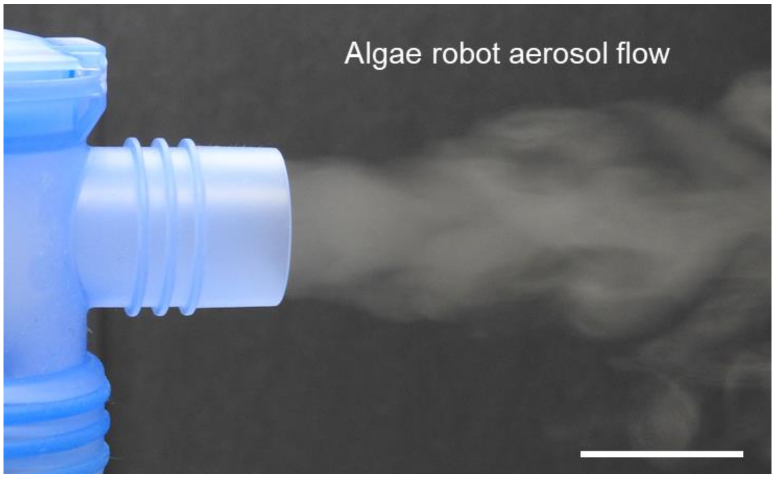

Supplementary Fig. 5. Representative image of algae robot aerosol flow from Supplementary Video 2. Scale bar, 1 cm.

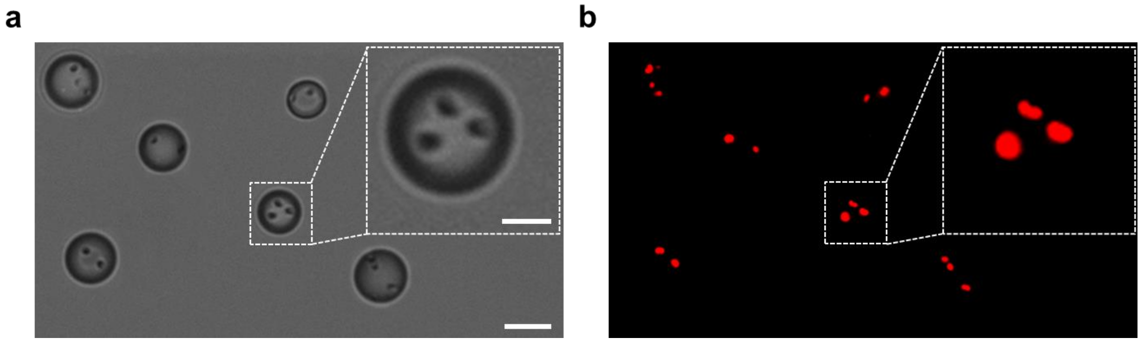

Supplementary Fig. 6. Fluorescence microscopy images of algae robot aerosol particles. Brightfield (a) and Cy5 (b) fluorescence images showing aerosol particles loaded with algae robots. Scale bars, 5 µm (main) and 2 µm (zoomed-in).

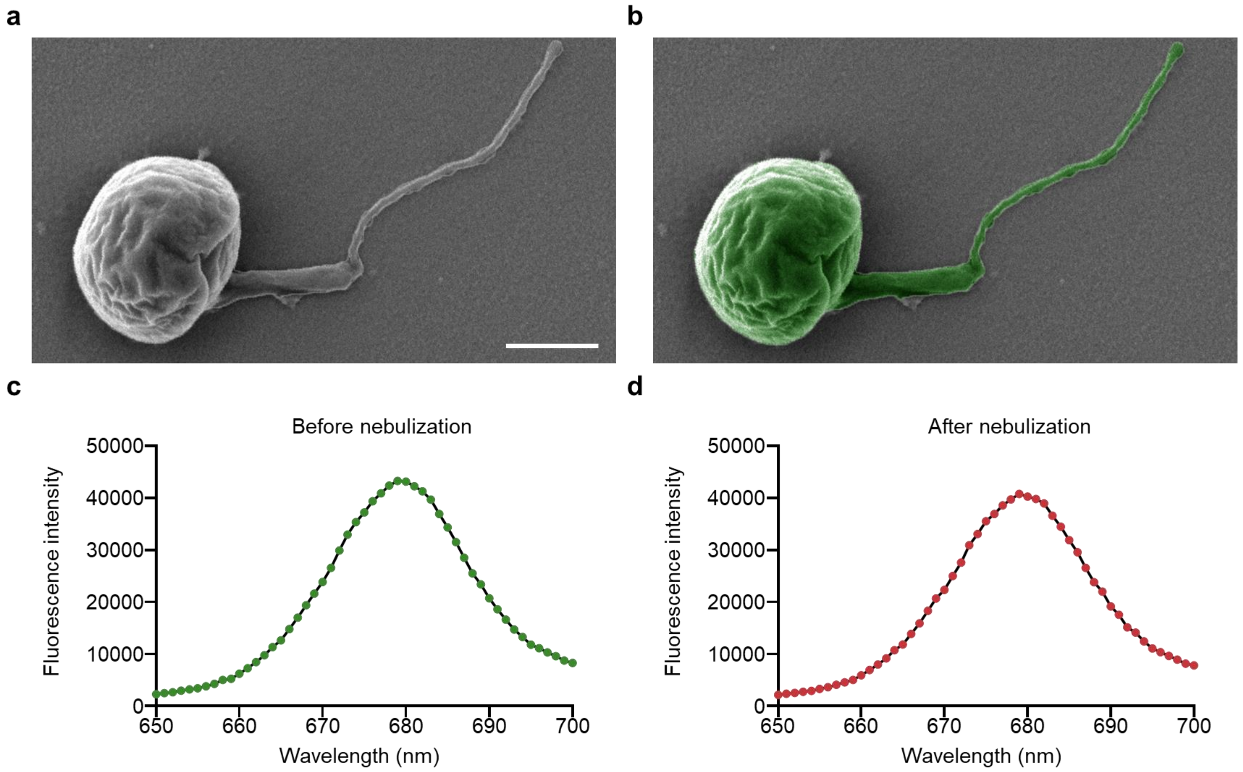

Supplementary Fig. 7. Characterization of algae robot morphology with nebulization. a,b, Representative SEM (a) and pseudocolored SEM (b) images of an algae robot post nebulization. Scale bar, 500 nm. **c,d,** The optical spectra of algae robot in 1× PBS buffer before (c) and after (d) nebulization at 22 °C.

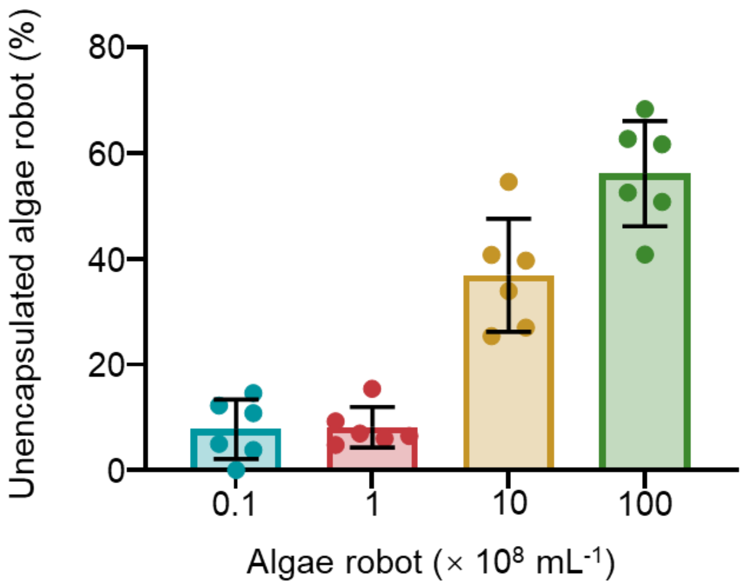

Supplementary Fig. 8. Encapsulation ratio of algae robot aerosol particles at different algae robot loadings of 1 × 10^7, 1 × 10^8, 1 × 10^9, and 1 × 10^{10} mL^{-1} with a constant system air flow rate of 4 L min^{-1} (n = 6, mean ± s.d.).

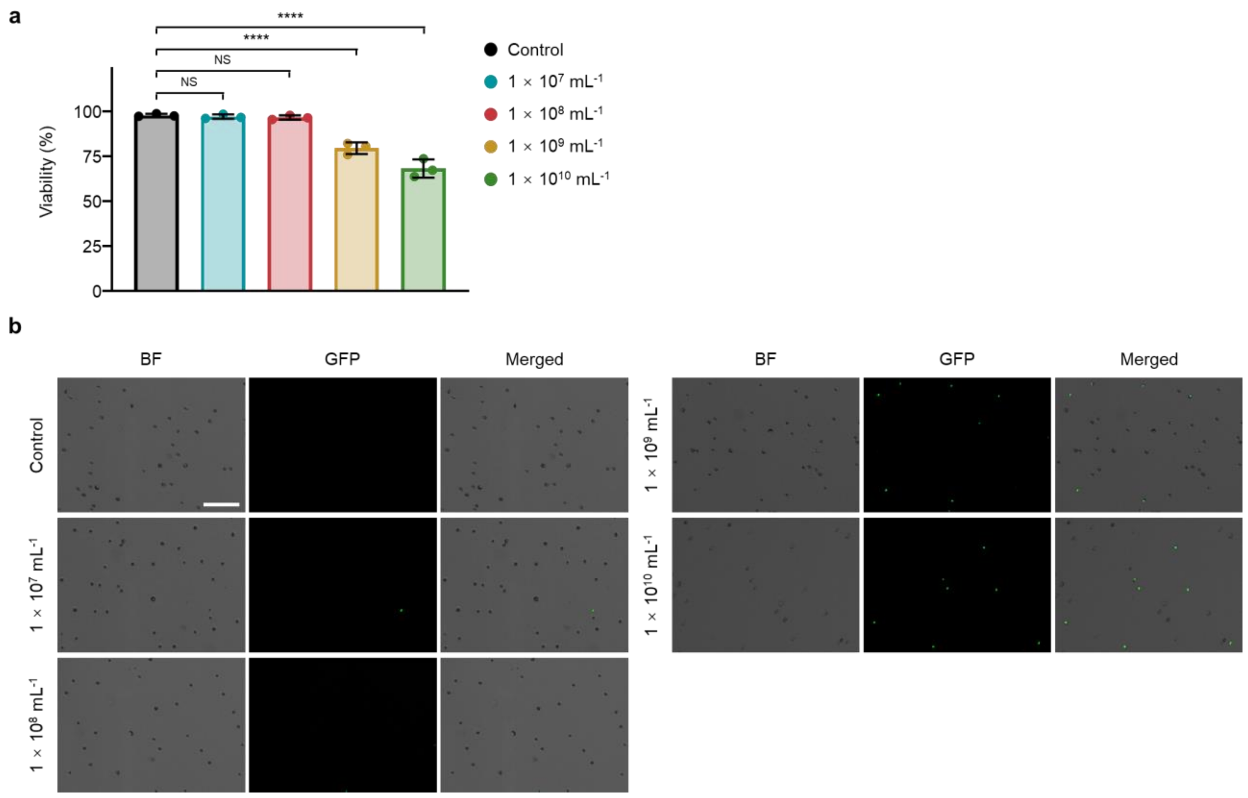

Supplementary Fig. 9. Viability of algae robots after nebulization. a, Comparison of the viability ratio of algae robots at different loadings of 1×10^7, 1×10^8, 1×10^9, and 1×10^{10} mL^{-1} with a constant system air flow rate of 4 L min^{-1} after nebulization versus the algae robot before nebulization (control) in SLF at 22 °C (n = 3, mean ± s.d.). **b,** Representative fluorescence microscopy images of algae robots before and after nebulization in SLF at 22 °C. Green channel (GFP): SYTOX-labeled dead algae. Scale bar, 10 µm. Statistical analysis for the viability ratio of algae robot was performed using repeated-measure one-way analysis of variance (ANOVA). NS: $P > 0.05$, ****$P \leq 0.0001$.

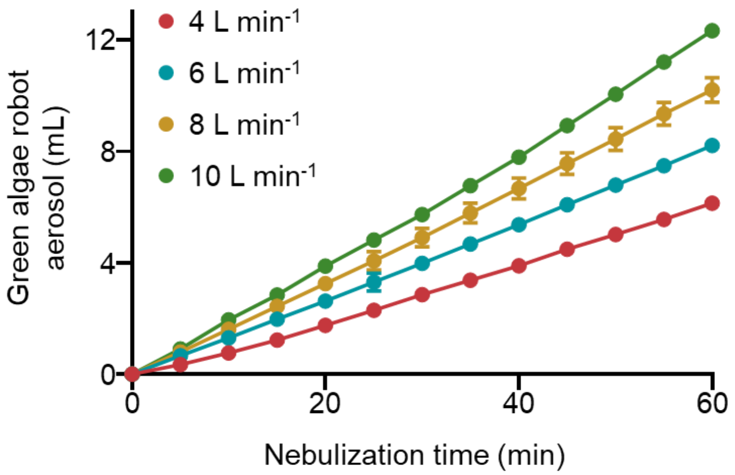

Supplementary Fig. 10. The dosage rate of the algae robot nebulizer with different system air flow rates of 4, 6, 8, and 10 L min^{-1} at the same algae robot loading of 1 × 10^{8} mL^{-1} (n = 3, mean ± s.d.).

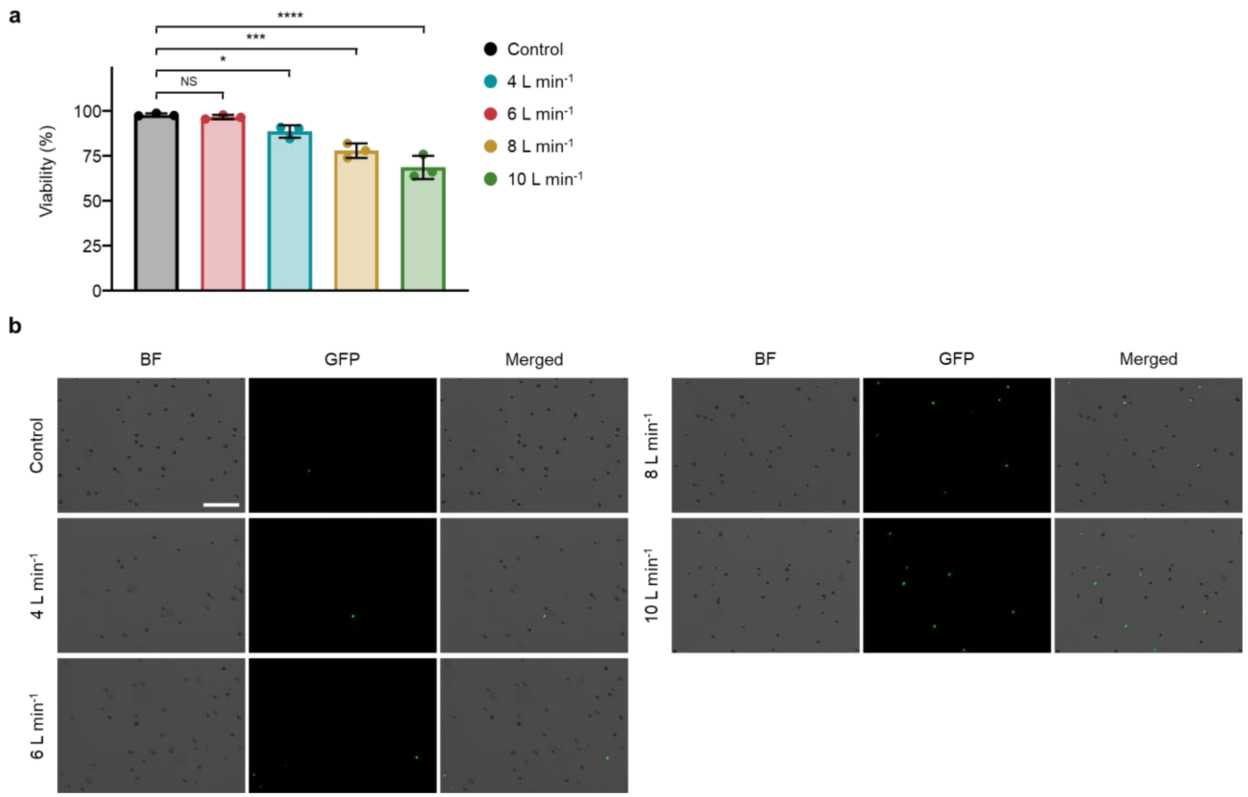

Supplementary Fig. 11. Impact of flow rate on algae robot viability after nebulization. a, The viability of algae robots with different system air flow rates of 4, 6, 8, and 10 L min^{-1} at the same algae robot loading of 1 × 10^{8} mL^{-1} after nebulization versus the algae robots before nebulization (control) in SLF at 22 °C (n = 3, mean ± s.d.). **b,** Representative microscopy fluorescence images of algae robots before and after nebulization in SLF at 22 °C. Green channel (GFP): SYTOX-labeled dead algae. Scale bar, 10 μm. Statistical analysis for the viability ratio of algae robot was performed using repeated-measure one-way analysis of variance (ANOVA). NS: $P > 0.05$, *$P \leq 0.05$, ***$P \leq 0.001$, ****$P \leq 0.0001$.

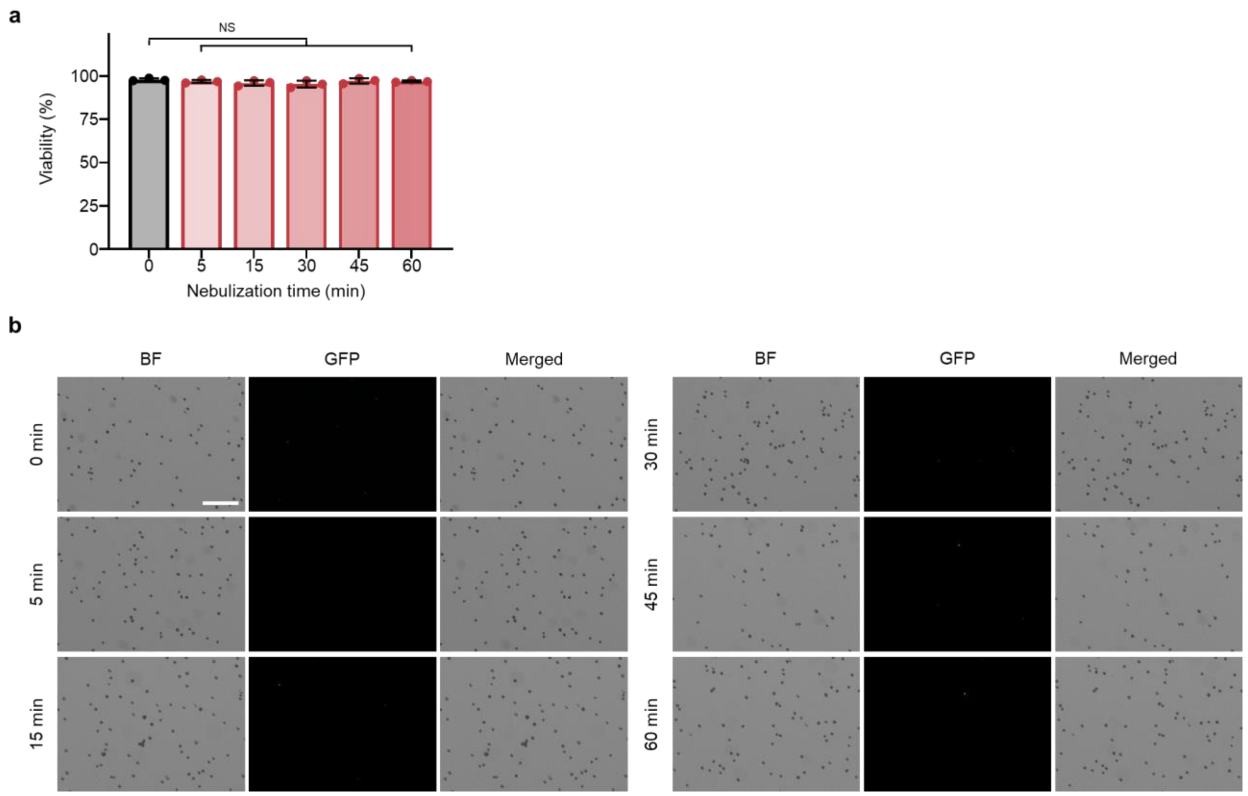

Supplementary Fig. 12. Impact of nebulization time on algae robot viability after nebulization. **a,** Viability of algae robots with a loading of 1×10^8 mL^{-1} and system air flow rate of 4 L min^{-1} after different nebulization times of 5, 15, 30, 45, and 60 min versus the algae robots before nebulization (0 min) in SLF at 22 °C (n = 3, mean ± s.d.). **b,** Representative microscopy fluorescence images of algae robots before and after nebulization in SLF at 22 °C. Green channel (GFP): SYTOX-labeled dead algae. Scale bar, 10 µm. Statistical analysis for the viability ratio of algae robot was performed using repeated-measure one-way analysis of variance (ANOVA). NS: $P > 0.05$.

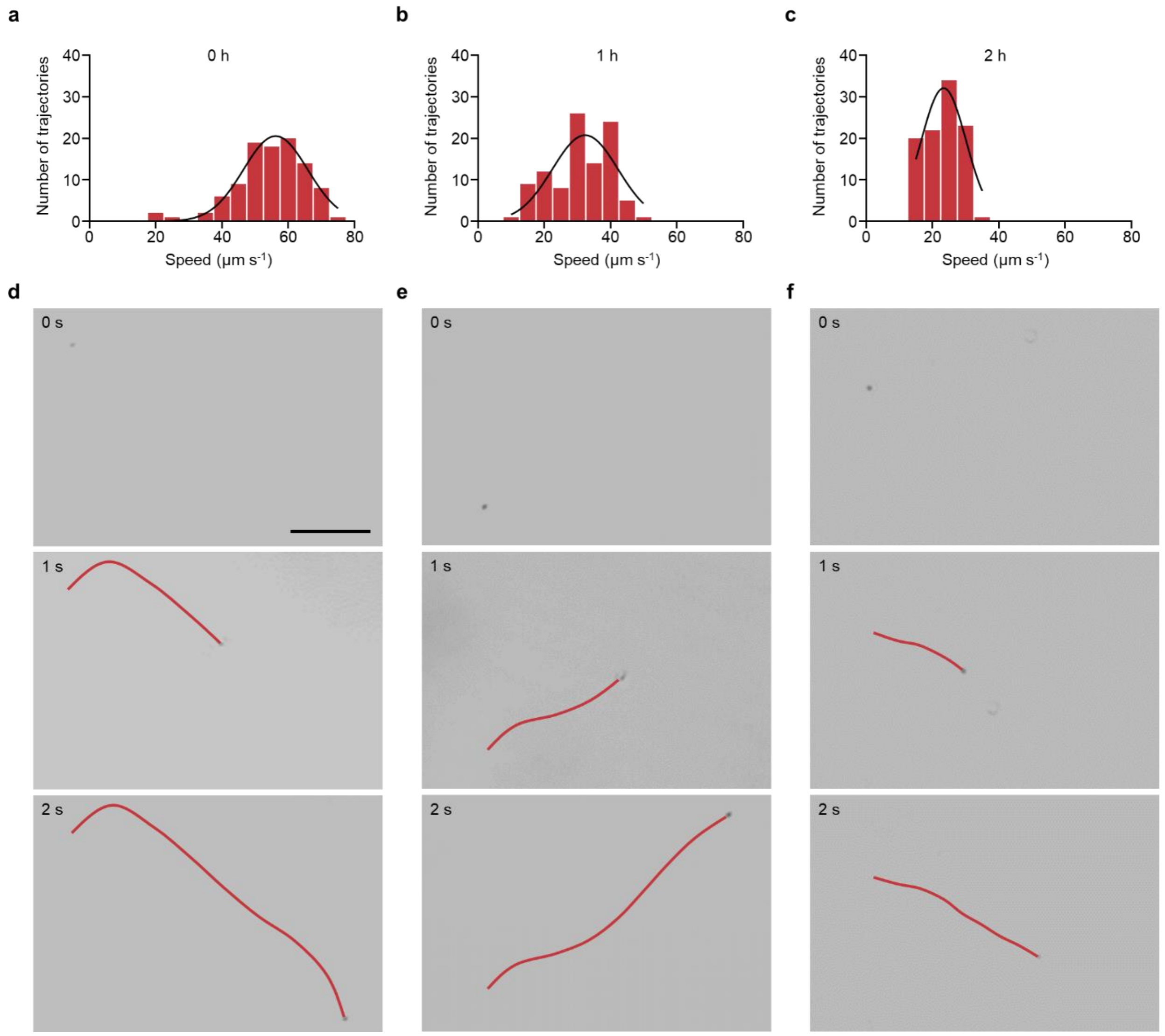

Supplementary Fig. 13. Speed distribution and representative tracking of algae robots after nebulization at 37 °C. a-c, Algae robot speed distribution in SLF at 0 h (a), 1 h (b), and 2 h (c) at 37 °C (n = 100). **d-f,** Representative trajectories corresponding to 0, 1, and 2 s, at 0 h (d), 1 h (e), and 2 h (f) at 37 °C. Scale bar, 20 μm.

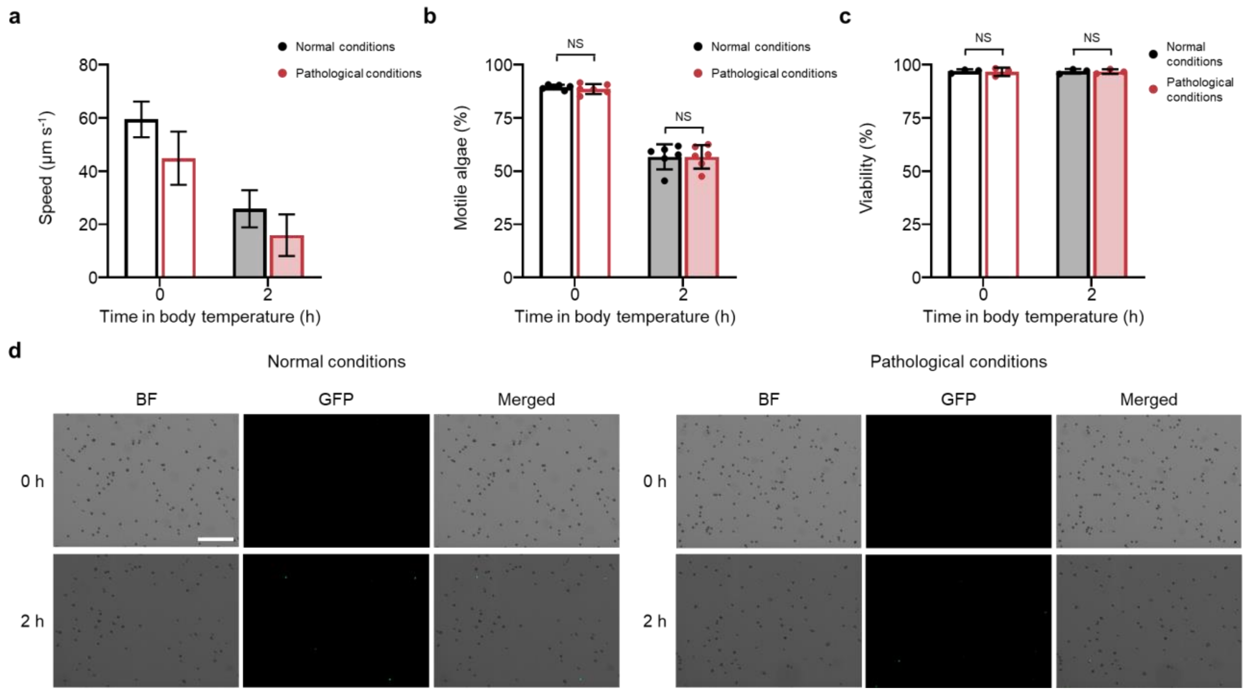

Supplementary Fig. 14. Motility characterization of algae robots in pathological conditions. a, Speed of the algae robot in normal conditions (pure SLF) and pathological conditions (with mucus and cytokines at pH=6) at 0 h and 2 h at 37 °C (n = 100, mean ± s.d.). **b,c,** Motility ratio (b, n = 6, mean ± s.d.) and viability (c, n = 3, mean ± s.d.) of the algae robot in normal conditions (pure SLF) and pathological conditions (with mucus and cytokines at pH=6) at 0 h and 2 h at 37 °C. **d,** Representative fluorescence microscopy images of algae robots in normal conditions (pure SLF) and pathological conditions (with mucus and cytokines at pH=6) at 0 h and 2 h at 37 °C. Green channel (GFP): SYTOX-labeled dead algae. Scale bar, 10 μm. Statistical analysis for the motility and viability ratio of algae robots was performed using unpaired two-tailed *t*-test. NS: $P > 0.05$.

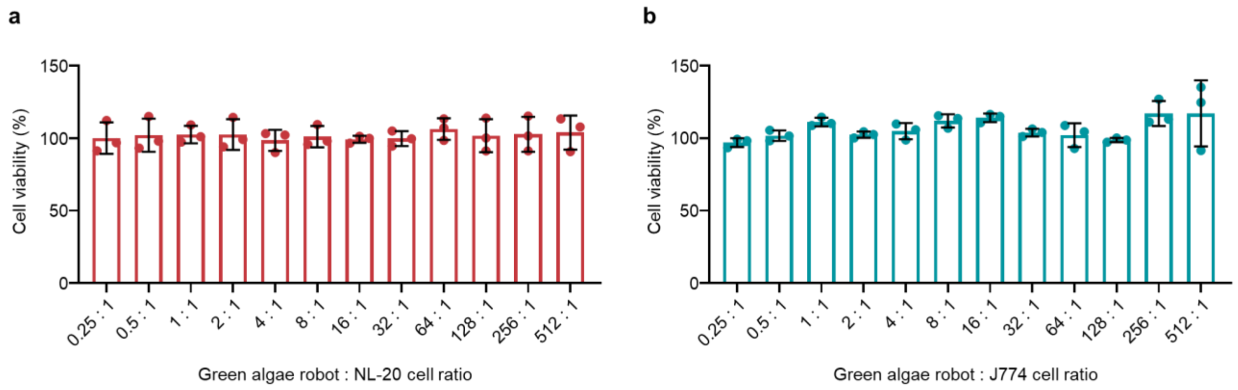

Supplementary Fig. 15. Cytotoxicity of algae robots. a,b, Algae robots were incubated with human lung epithelial cells (NL-20, a) and macrophages (J774, b) at different ratios for 24 h at 37 °C, then the cell viability was evaluated using a CellTiter Aqueous One Solution cell proliferation assay (MTS) (n = 3, mean ± s.d.).

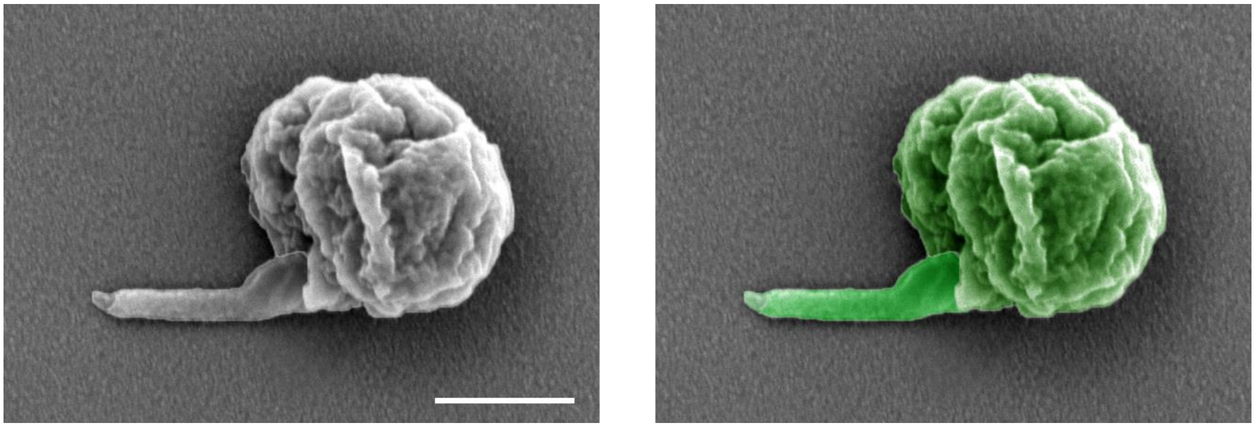

Supplementary Fig. 16. Representative SEM and pseudo-colored SEM images of static algae (without flagella). Scale bar, 500 nm.

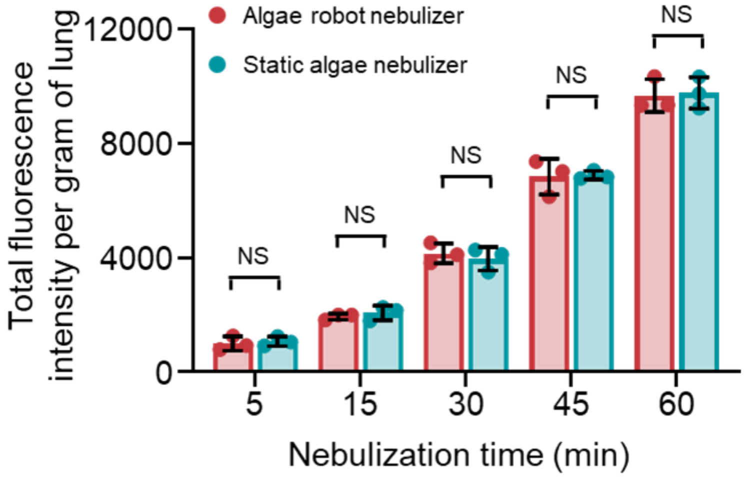

Supplementary Fig. 17. Total fluorescence intensity per gram of lung tissue after delivery of algae robots and static algae via nebulization for increasing periods of time (n = 3, mean ± s.d.). Statistical analysis for the fluorescence intensity was performed using unpaired two-tailed *t*-test. NS: $P > 0.05$.

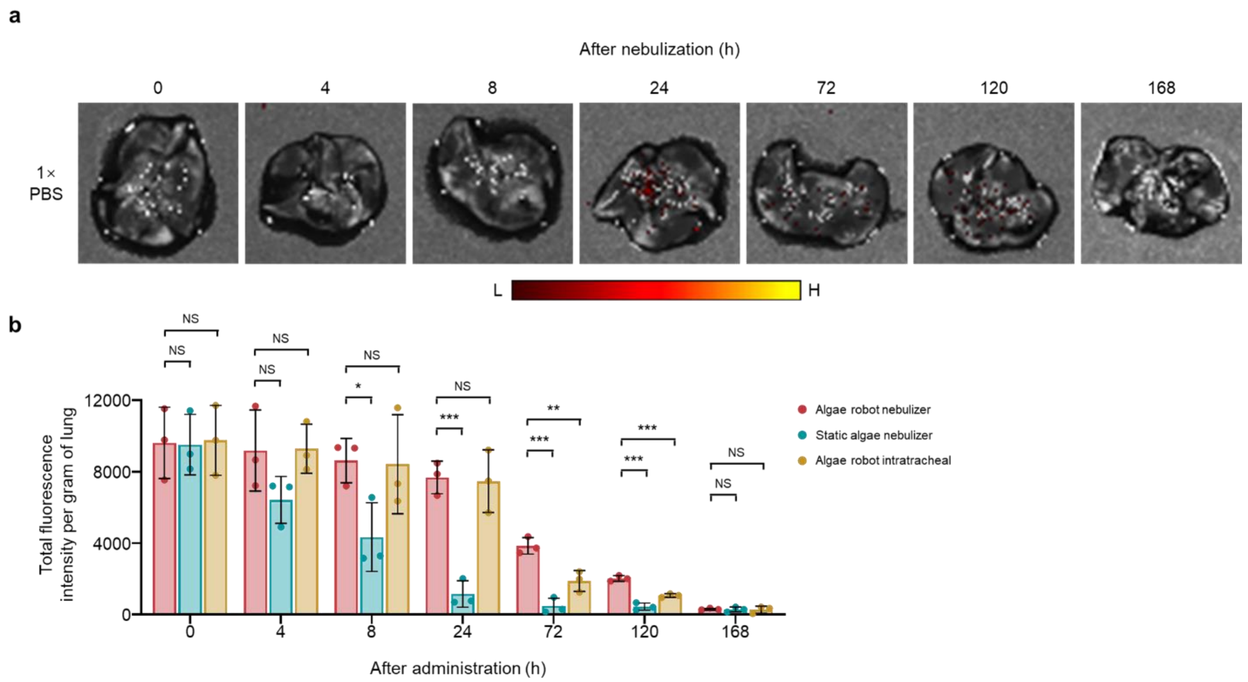

Supplementary Fig. 18. a, Ex vivo fluorescent images of lungs at various timepoints after 60 min of nebulization administration with 1× PBS. H, high signal; L, low signal. **b,** Fluorescence intensity per gram of lung samples after delivery of algae robots and static algae via nebulization, as well as algae robots via intratracheal administration (n = 3, mean ± s.d.). Statistical analysis for the fluorescence intensity was performed using repeated-measure one-way analysis of variance (ANOVA). NS: $P > 0.05$, $^*P \leq 0.05$, $^{**}P \leq 0.01$, $^{***}P \leq 0.001$.

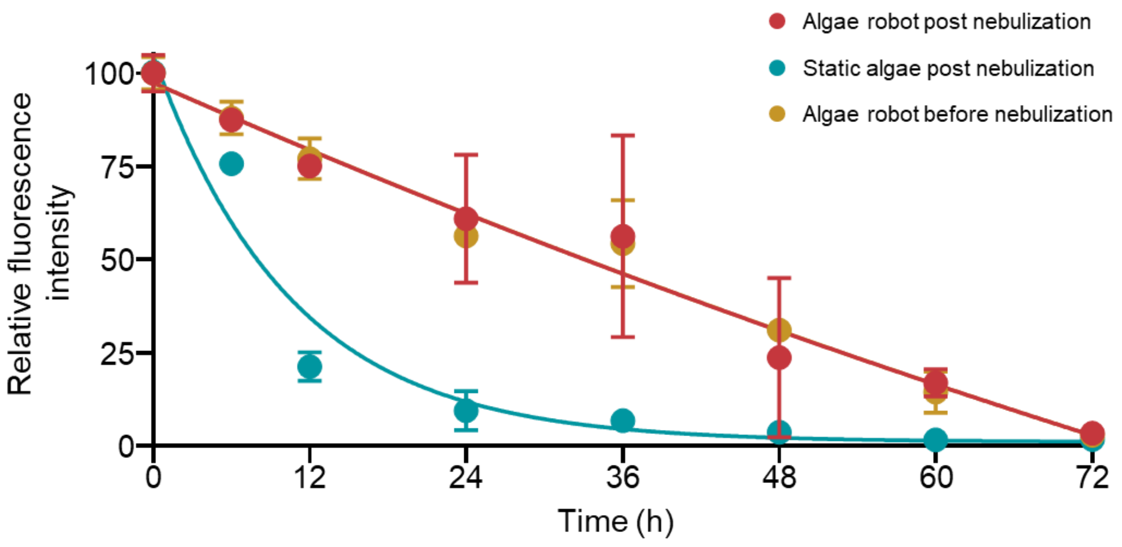

Supplementary Fig. 19. Relative fluorescence intensity of the algae robot post-nebulization, static algae post-nebulization and algae robot before nebulization over time after incubation with macrophage cells *in vitro* (n = 3, mean ± s.d.).

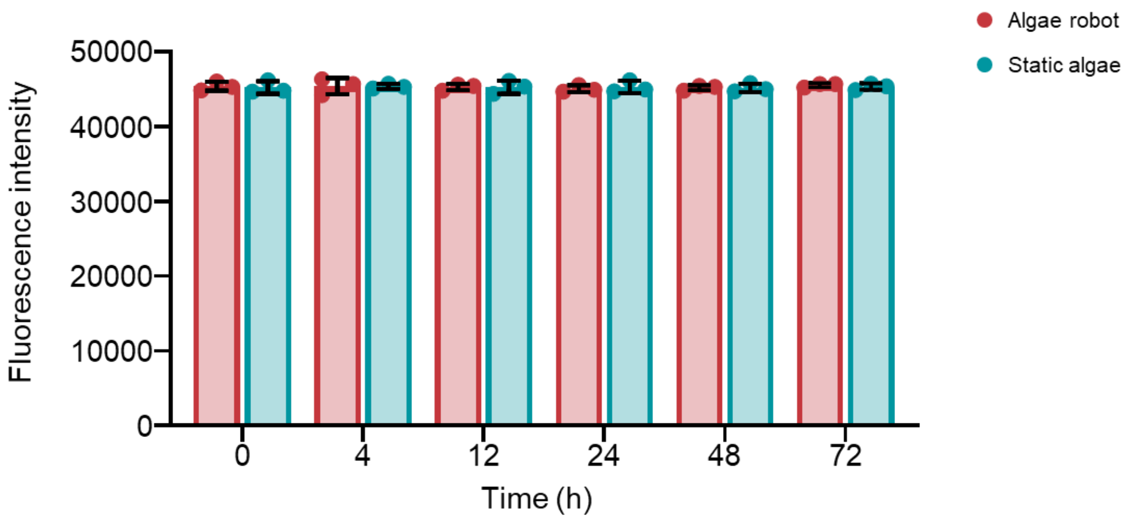

Supplementary Fig. 20. Fluorescence intensity of algae robots and static algae in SLF at 37 °C for up to 72 h without light exposure (n = 3, mean ± s.d.).

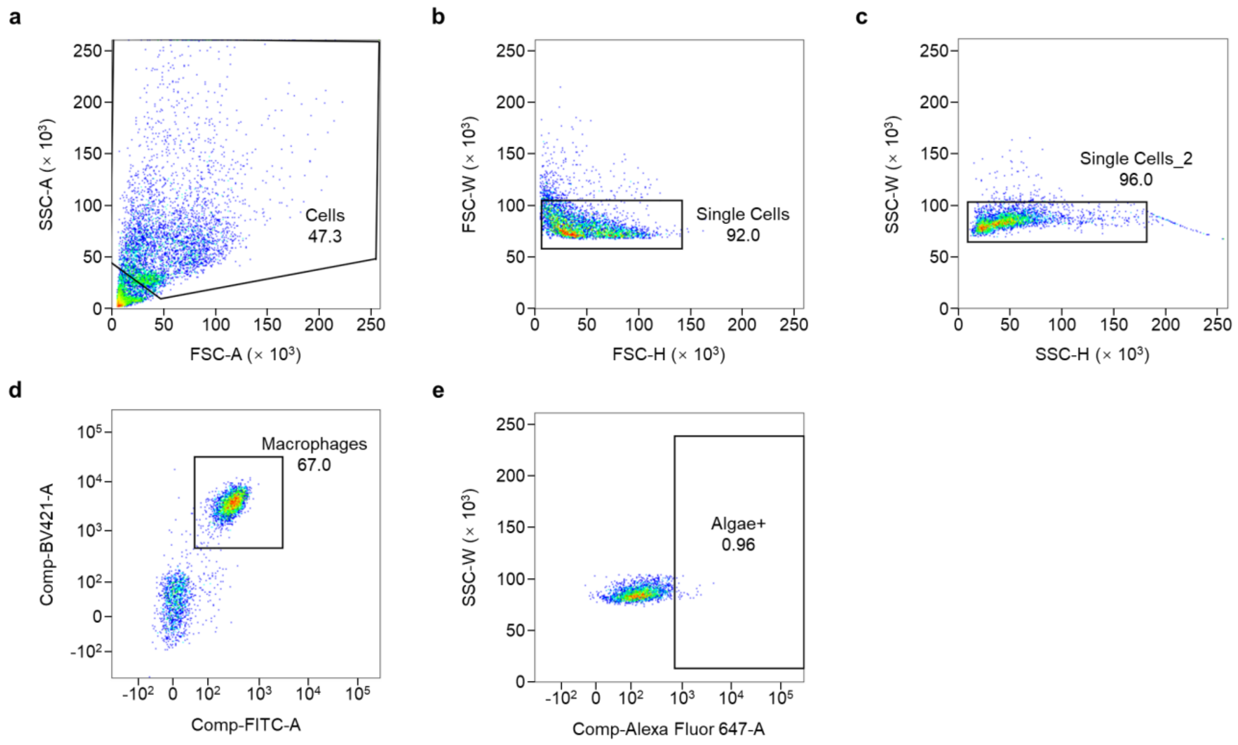

Supplementary Fig. 21. Flow cytometry analysis of gating strategy (from **a** to **e**) for quantitation of alveolar macrophage ($CD11c^{+}$ Siglec-F^{+}) uptake of algae robots or static algae.

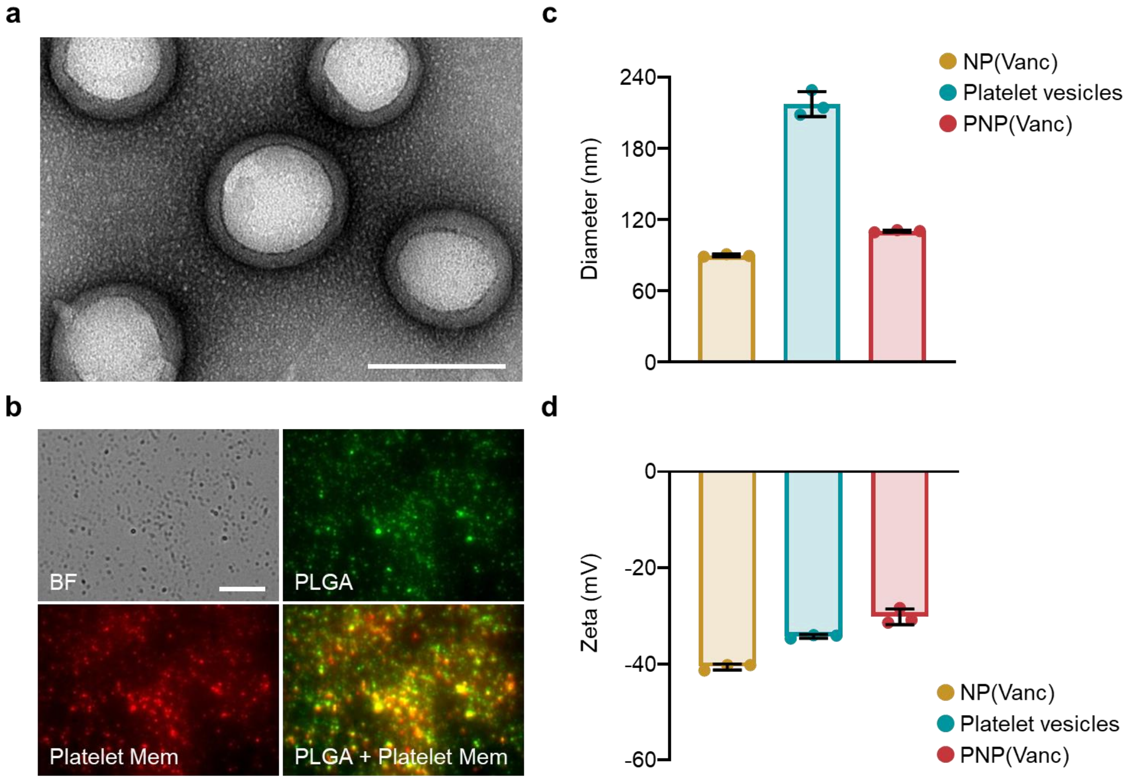

Supplementary Fig. 22. Characterization of platelet membrane-coated vancomycin-loaded PLGA nanoparticles (PNP(Vanc)). a, Representative TEM image of PNP(Vanc). Scale bar, 100 nm. **b,** Brightfield and fluorescent images of PNP(Vanc). DiO-labeled PLGA core in the GFP channel; DiI-labeled platelet membrane in the RFP channel. Scale bar, 1 μm. **c,d,** Hydrodynamic size (diameter) (c) and surface zeta potential (d) of vancomycin-loaded PLGA nanoparticles (NP(Vanc)), platelet vesicles, and PNP(Vanc) as measured by dynamic light scattering (DLS) (n = 3, mean ± s.d.).

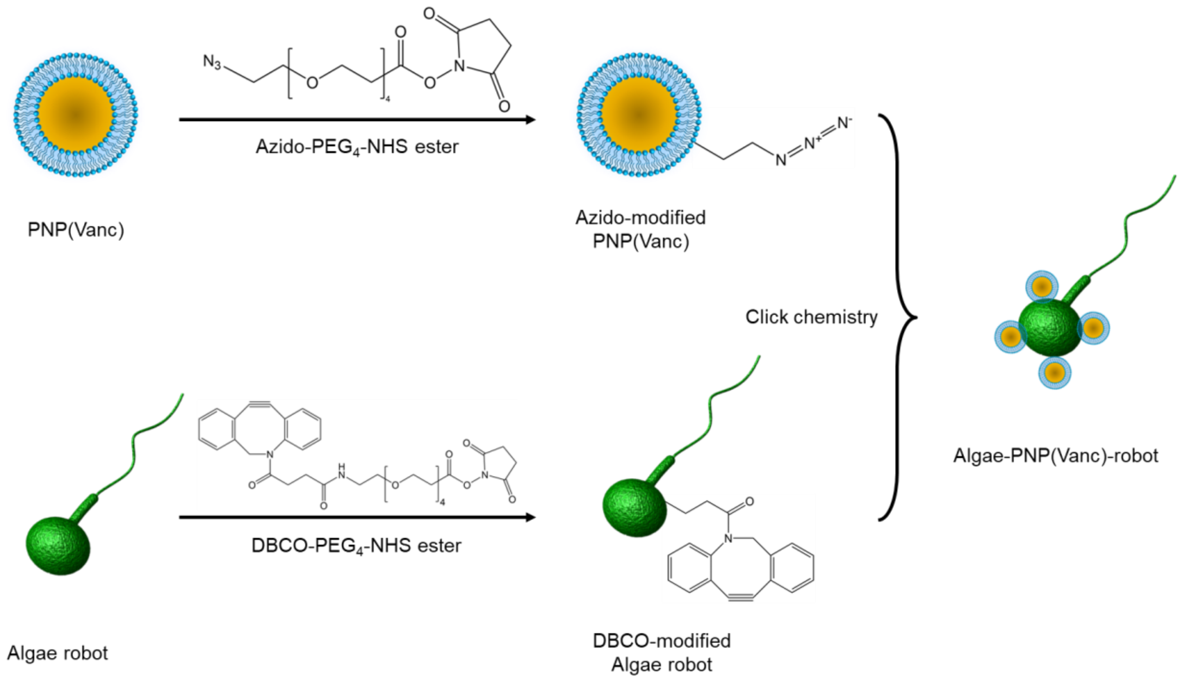

Supplementary Fig. 23. Modification of algae robots with PNP(Vanc) (denoted as 'algae-PNP(Vanc)-robot') through click chemistry.

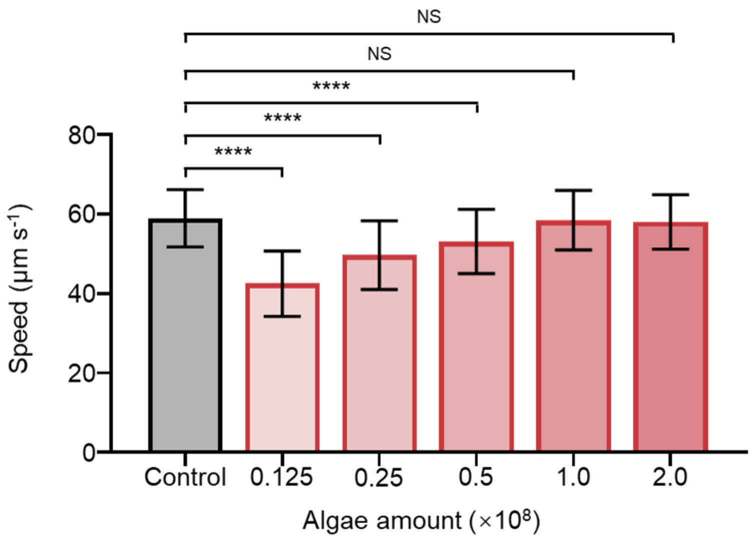

Supplementary Fig. 24. Speed of algae robots at different amounts (0.125, 0.25, 0.5, 1, and 2 × 10^8) mixed with 1 mg of PNP(Vanc) (n = 100, mean ± s.d.). Statistical analysis for the motion speed was performed using repeated-measure one-way analysis of variance (ANOVA). NS: $P > 0.05$, ****$P \leq 0.0001$.

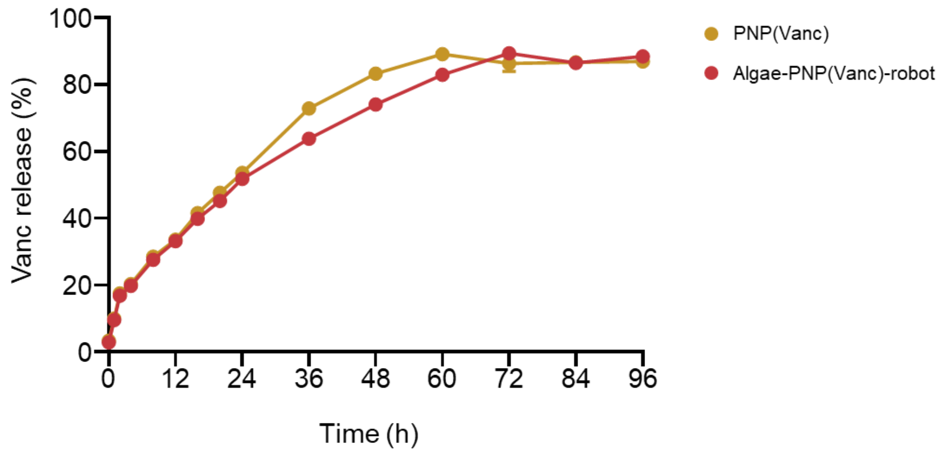

Supplementary Fig. 25. Cumulative drug release profile of PNP(Vanc) and algae-PNP(Vanc)-robot in SLF at 37 °C (n = 3, mean ± s.d.).

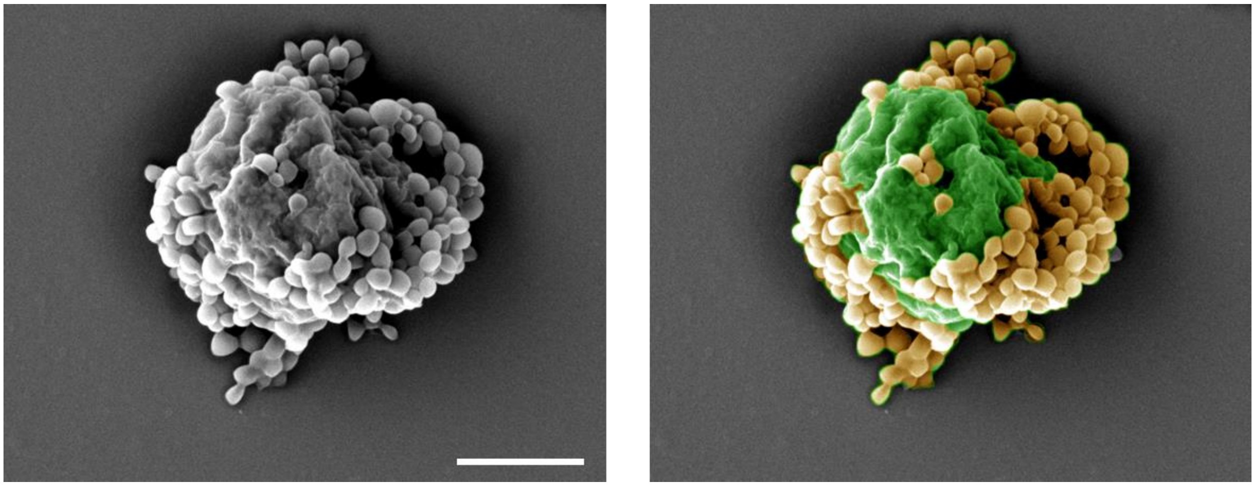

Supplementary Fig. 26. Representative SEM and pseudo-colored SEM images of static algae-PNP(Vanc) (without flagella). Scale bar, 500 nm.

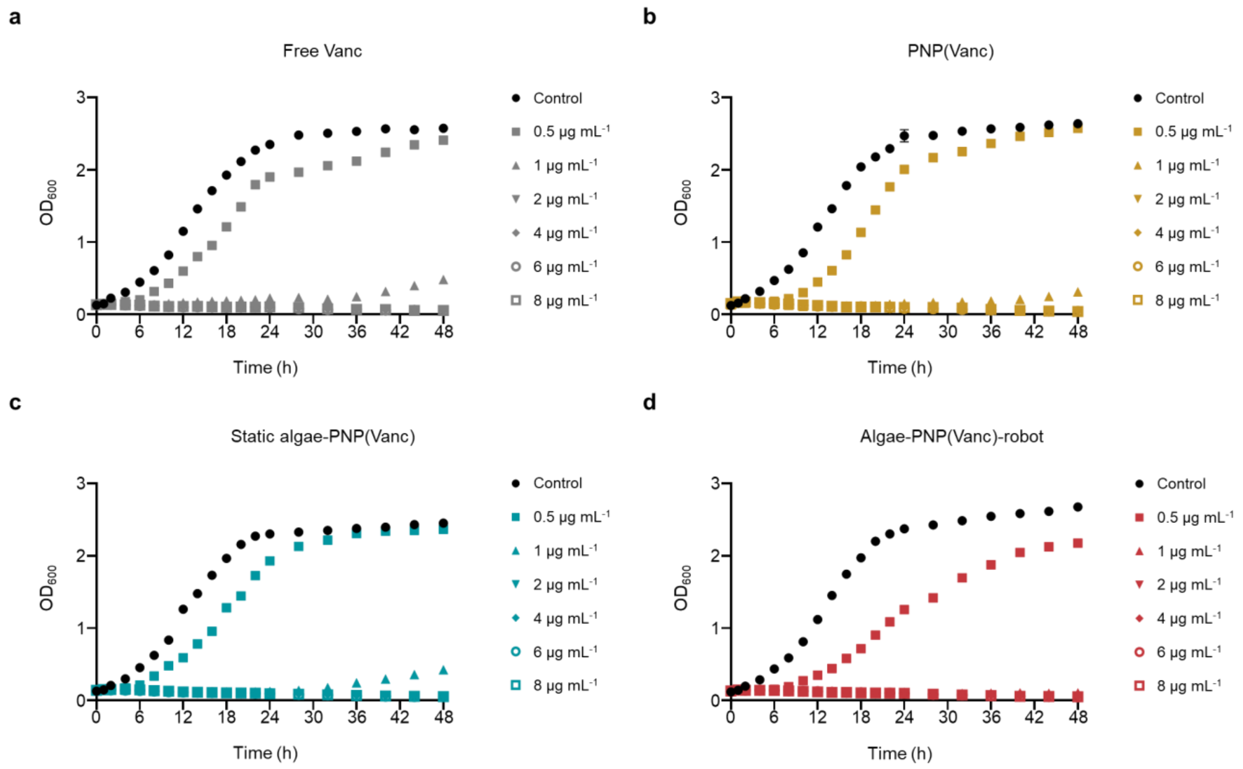

Supplementary Fig. 27. Growth curves of methicillin-resistant *Staphylococcus aureus* (MRSA). a-d, Time-dependent OD_{600} of MRSA incubated with free Vanc (a), PNP(Vanc) (b), static algae-PNP(Vanc) (c), and algae-PNP(Vanc)-robot (d) at various drug dosages at body temperature (37 °C) (n = 3, mean ± s.d.).

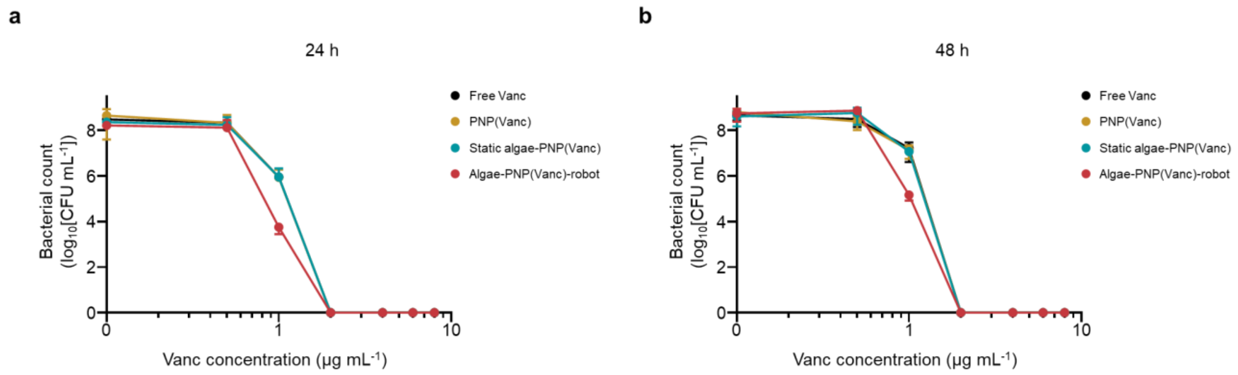

Supplementary Fig. 28. *In vitro* bactericidal activity against MRSA. a,b, *In vitro* bacterial counts after incubation with free Vanc, PNP(Vanc), static algae-PNP(Vanc), and algae-PNP(Vanc)-robot at various drug dosages for 24 h (a) and 48 h (b) (n = 3, geometric mean ± s.d.).

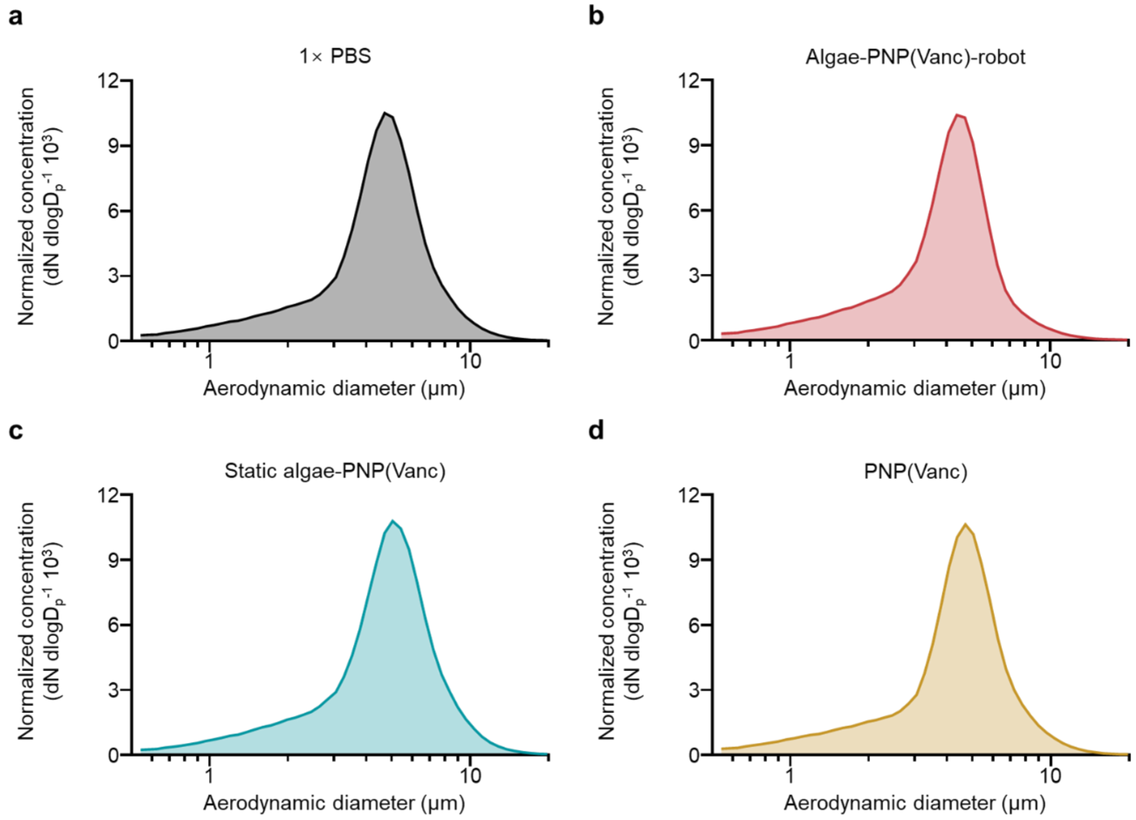

Supplementary Fig. 29. Aerodynamic size distribution of aerosol particles. a-d, Aerodynamic size distribution of aerosol particles with 1× PBS (a), algae-PNP(Vanc)-robot (b), static algae-PNP(Vanc) (c), and PNP(Vanc) (d) at a loading concentration of 1×10^{8} mL^{-1} algae, corresponding to 10 μg mL^{-1} of Vanc, nebulized at a system air flow rate of 4 L min^{-1}.

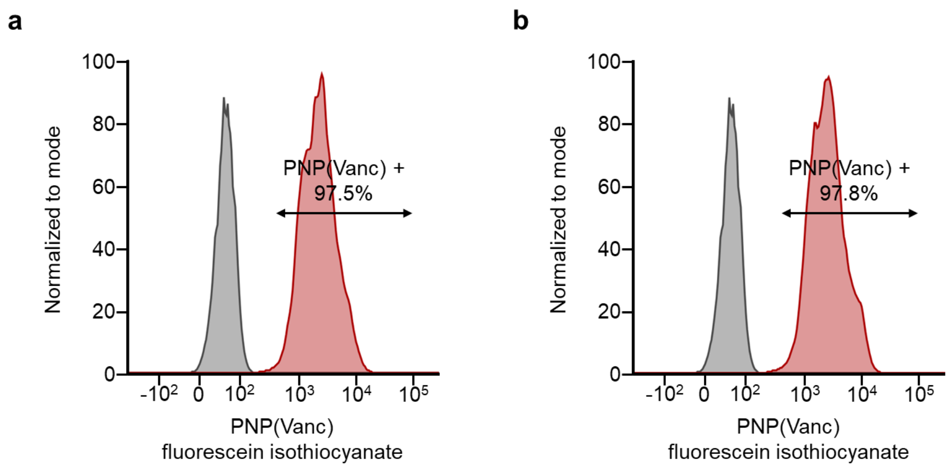

Supplementary Fig. 30. Algae-PNP(Vanc)-robot conjugation stability after nebulization. a,b, Representative flow cytometry histograms of unmodified algae robots (black) and algae-PNP(Vanc)-robot fabricated with DiO-labeled PNP(Vanc) (red) before (a) and after (b) nebulization.

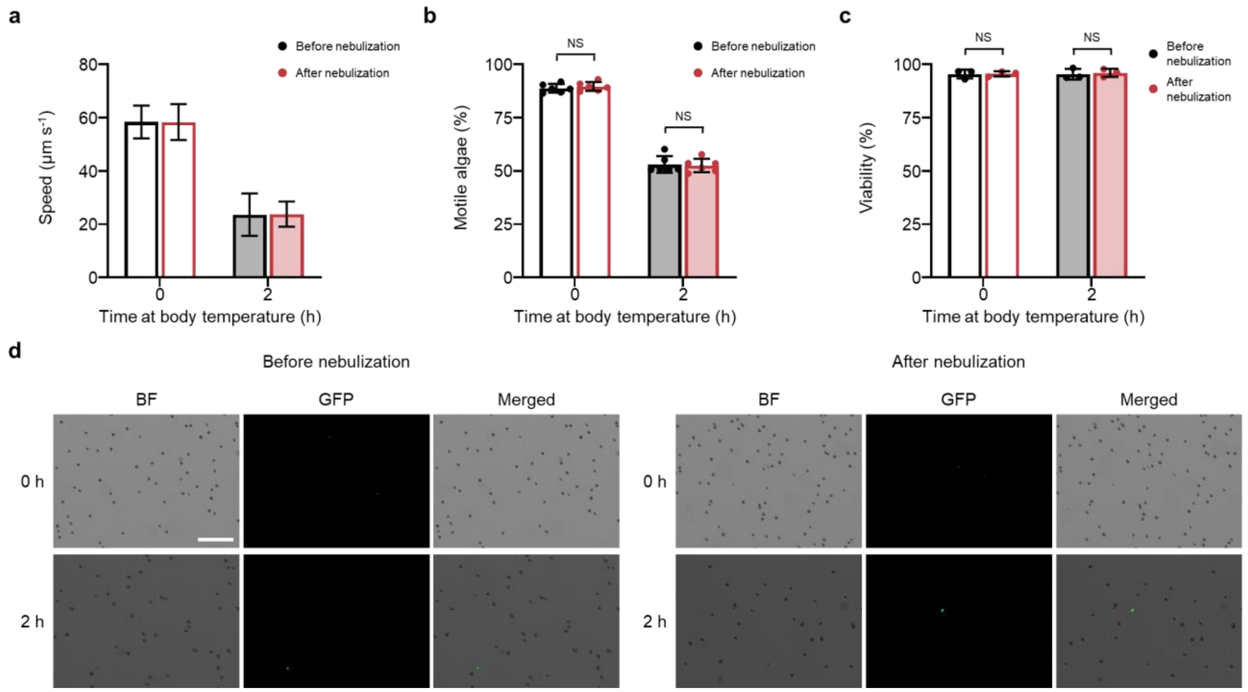

Supplementary Fig. 31. Motility of algae-PNP(Vanc)-robot with nebulization. a, Speed of algae-PNP(Vanc)-robot before and after nebulization in SLF at 0 h and 2 h at 37 °C (n = 100, mean ± s.d.). **b,c,** Motility ratio (b, n = 6, mean ± s.d.) and viability (c, n = 3, mean ± s.d.) of algae-PNP(Vanc)-robot in SLF before and after nebulization at 0 h and 2 h at 37 °C. **d,** Representative fluorescence microscopy images of algae-PNP(Vanc)-robot before and after nebulization in SLF at 0 h and 2 h at 37 °C. Green channel (GFP): SYTOX-labeled dead algae. Scale bar, 10 μm. Statistical analysis for the motility and viability ratio of algae-PNP(Vanc)-robot was performed using unpaired two-tailed *t*-test. NS: $P > 0.05$.

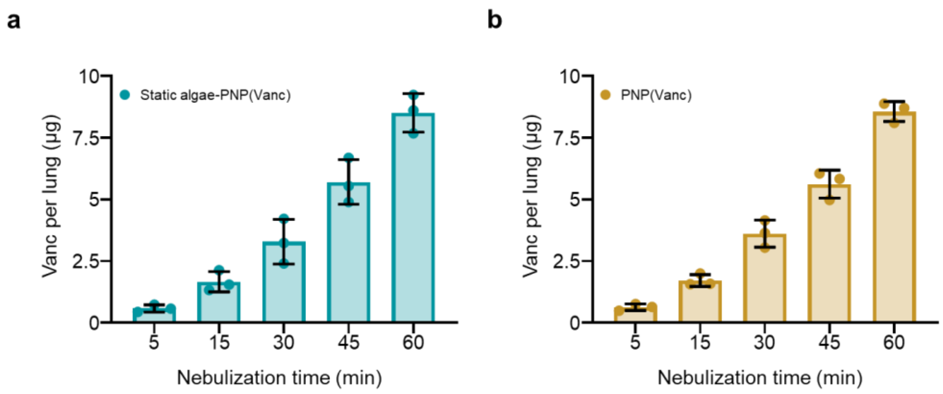

Supplementary Fig. 32. a,b, Quantification of Vanc inhaled per lung after different nebulization times with static algae-PNP(Vanc) (a) and PNP(Vanc) (b) (n = 3, mean ± s.d.).

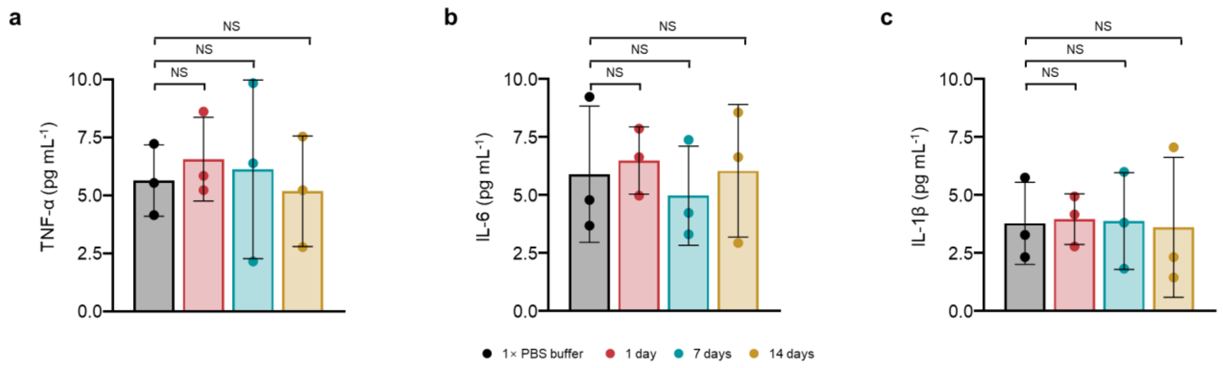

Supplementary Fig. 33. Cytokine levels measured on days 1, 7 and 14 post-administration. a-c, Concentration of cytokines (TNF-α, IL-6, and IL-1β) in mouse blood (n = 3, mean ± s.d.). Statistical analysis for the cytokine levels was performed using repeated-measure one-way analysis of variance (ANOVA). NS: $P > 0.05$.

Supplementary Table

Table 1. Composition of simulated lung fluid (SLF).

Component	Concentration
Albumin	8.8 mg mL^{-1}
Ascorbate	140 μM
Cholesterol	0.1 mg mL^{-1}
DPPC	4.8 mg mL^{-1}
DPPG	0.5 mg mL^{-1}
Gentamicin	1 μL mL^{-1}
Glutathione	170 μM
HBSS	77.5 μL mL^{-1}
IgG	2.6 mg mL^{-1}
Transferrin	1.5 mg mL^{-1}
Urate	95 μM

Supplementary Videos

Supplementary Video 1. Motion of algae robots in various media at 22 °C.

Supplementary Video 2. Aerosol flow of the algae-based biohybrid microrobot nebulizer system at 22 °C.

Supplementary Video 3. Motion of algae robots in aerosol particles at 22 °C.

Supplementary Video 4. Motion of algae robots with various loadings in SLF at 22 °C post nebulization.

Supplementary Video 5. Motion of algae robots with various system air flow rates in SLF at 22 °C post nebulization.

Supplementary Video 6. Representative 2-s tracking of algae robots in SLF at 37 °C at 0, 1, and 2 h post nebulization.

Supplementary Video 7. Aerosol administration to mice using the algae-based biohybrid microrobot nebulizer system at 22 °C.

Supplementary Video 8. Motion of algae-PNP(Vanc)-robot in simulated lung fluid at 37 °C at 0 and 2 h post nebulization.
