# Supplementary material for: Inhalable biohybrid microrobots: a non-invasive approach for lung treatment
Source: Nat Commun. 2025 Jan 14;16:666. doi: 10.1038/s41467-025-56032-4 (PMC11733022; doi:10.1038/s41467-025-56032-4)
Supplement: Supplementary file 2 — Description of Additional Supplementary Files [file 41467_2025_56032_MOESM2_ESM.pdf]

Description of Additional Supplementary Files

File Name: Supplementary Video 1
Description: Motion of algae robots in various media at 22 °C.

File Name: Supplementary Video 2
Description: Aerosol flow of the algae-based biohybrid microrobot nebulizer system at 22 °C.

File Name: Supplementary Video 3
Description: Motion of algae robots in aerosol particles at 22 °C.

File Name: Supplementary Video 4
Description: Motion of algae robots with various loadings in SLF at 22 °C post nebulization.

File Name: Supplementary Video 5
Description: Motion of algae robots with various system air flow rates in SL at 22 °C post nebulization.

File Name: Supplementary Video 6
Description: Representative 2-s tracking of algae robots in SLF at 37 °C at 0, 1 and 2 h post nebulization.

File Name: Supplementary Video 7
Description: Aerosol administration to mice using the algae-based biohybrid microrobot nebulizer system at 22 °C

File Name: Supplementary Video 8
Description: Motion of algaePNP(Vanc)-robot in simulated lung fluid at 37 °C at 0 and 2 h post nebulization.
