# Supplementary material for: Inhalable biohybrid microrobots: a non-invasive approach for lung treatment
Source: Nat Commun. 2025 Jan 14;16:666. doi: 10.1038/s41467-025-56032-4 (PMC11733022; doi:10.1038/s41467-025-56032-4)
Supplement: Supplementary file 11 — Reporting Summary [file 41467_2025_56032_MOESM11_ESM.pdf]

nature portfolio

Corresponding author(s): Joseph Wang
Liangfang Zhang

Last updated by author(s): Dec 23, 2024

Reporting Summary

Nature Portfolio wishes to improve the reproducibility of the work that we publish. This form provides structure for consistency and transparency in reporting. For further information on Nature Portfolio policies, see our Editorial Policies and the Editorial Policy Checklist.

Statistics

For all statistical analyses, confirm that the following items are present in the figure legend, table legend, main text, or Methods section.

n/a	Confirmed	
☐	☒	The exact sample size (*n*) for each experimental group/condition, given as a discrete number and unit of measurement
☐	☒	A statement on whether measurements were taken from distinct samples or whether the same sample was measured repeatedly
☐	☒	The statistical test(s) used AND whether they are one- or two-sided<br>*Only common tests should be described solely by name; describe more complex techniques in the Methods section.*
☐	☒	A description of all covariates tested
☐	☒	A description of any assumptions or corrections, such as tests of normality and adjustment for multiple comparisons
☐	☒	A full description of the statistical parameters including central tendency (e.g. means) or other basic estimates (e.g. regression coefficient) AND variation (e.g. standard deviation) or associated estimates of uncertainty (e.g. confidence intervals)
☐	☒	For null hypothesis testing, the test statistic (e.g. *F*, *t*, *r*) with confidence intervals, effect sizes, degrees of freedom and *P* value noted<br>*Give P values as exact values whenever suitable.*
☒	☐	For Bayesian analysis, information on the choice of priors and Markov chain Monte Carlo settings
☒	☐	For hierarchical and complex designs, identification of the appropriate level for tests and full reporting of outcomes
☒	☐	Estimates of effect sizes (e.g. Cohen's *d*, Pearson's *r*), indicating how they were calculated

Our web collection on statistics for biologists contains articles on many of the points above.

Software and code

Policy information about availability of computer code

Data collection	No standalone or custom software was used.
Data analysis	Software used in analysis include GraphPad Prism 10, FlowJo 7.6, ImageJ, and Microsoft Excel.

For manuscripts utilizing custom algorithms or software that are central to the research but not yet described in published literature, software must be made available to editors and reviewers. We strongly encourage code deposition in a community repository (e.g. GitHub). See the Nature Portfolio guidelines for submitting code & software for further information.

Data

Policy information about availability of data

All manuscripts must include a data availability statement. This statement should provide the following information, where applicable:

- Accession codes, unique identifiers, or web links for publicly available datasets
- A description of any restrictions on data availability
- For clinical datasets or third party data, please ensure that the statement adheres to our policy

All data supporting the findings of this study are available within the article and its supplementary files. Any additional requests for information can be directed to, and will be fulfilled by, the corresponding authors. Source data are provided with this paper.

Research involving human participants, their data, or biological material

Policy information about studies with human participants or human data. See also policy information about sex, gender (identity/presentation), and sexual orientation and race, ethnicity and racism.

Reporting on sex and gender	N/A.
Reporting on race, ethnicity, or other socially relevant groupings	N/A.
Population characteristics	N/A.
Recruitment	N/A.
Ethics oversight	N/A.

Note that full information on the approval of the study protocol must also be provided in the manuscript.

Field-specific reporting

Please select the one below that is the best fit for your research. If you are not sure, read the appropriate sections before making your selection.

[x] Life sciences [ ] Behavioural & social sciences [ ] Ecological, evolutionary & environmental sciences

For a reference copy of the document with all sections, see nature.com/documents/nr-reporting-summary-flat.pdf

Life sciences study design

All studies must disclose on these points even when the disclosure is negative.

Sample size	No sample size calculation was performed. The in vivo pneumonia treatment studies were evaluated with 6 mice per group for bacterial enumeration and survival studies. Sample sizes were determined based on prior experience with the animal model and pilot studies.
Data exclusions	No data were excluded.
Replication	Experiments were repeated and experimental results were reproducible.
Randomization	Samples were randomly allocated to different experimental groups before treatment. Organisms were cultured and maintained in the same environment and randomly allocated to each group.
Blinding	Investigators were not blinded to group allocation during data collection and analysis.

Reporting for specific materials, systems and methods

We require information from authors about some types of materials, experimental systems and methods used in many studies. Here, indicate whether each material, system or method listed is relevant to your study. If you are not sure if a list item applies to your research, read the appropriate section before selecting a response.

Materials & experimental systems

n/a	Involved in the study	
[ ]	[x]	Antibodies
[ ]	[x]	Eukaryotic cell lines
[x]	[ ]	Palaeontology and archaeology
[ ]	[x]	Animals and other organisms
[x]	[ ]	Clinical data
[x]	[ ]	Dual use research of concern
[x]	[ ]	Plants

Methods

n/a	Involved in the study	
[x]	[ ]	ChIP-seq
[ ]	[x]	Flow cytometry
[x]	[ ]	MRI-based neuroimaging

Antibodies

Antibodies used	Pacific Blue-conjugated anti-mouse CD11c (Biolegend, Cat. No. 117322, Clone N418), PE-conjugated anti-mouse Siglec-F (Biolegend, Cat. No. 155505, Clone S17007L).
Validation	Antibodies were validated by the manufacturer and used without further modifications.

Eukaryotic cell lines

Policy information about cell lines and Sex and Gender in Research

Cell line source(s)	Human platelet cells were collected from human type O- blood. Murine macrophage cells (J774A.1) and human bronchial epithelial cells (NL-20) were obtained from American Type Culture Collection (TIB-67 and CRL-2503).
Authentication	Cells were used without modification after receiving from the supplier and therefore were not authenticated.
Mycoplasma contamination	The cell line was tested monthly to be negative for mycoplasma contamination.
Commonly misidentified lines (See ICLAC register)	N/A.

Animals and other research organisms

Policy information about studies involving animals; ARRIVE guidelines recommended for reporting animal research, and Sex and Gender in Research

Laboratory animals	Male CD-1 mice were purchased from Charles River Labs.
Wild animals	N/A.
Reporting on sex	Male mice.
Field-collected samples	N/A.
Ethics oversight	All animal experiments were performed in accordance with NIH guidelines and approved by the Institutional Animal Care and Use Committee (IACUC) of University of California, San Diego.

Note that full information on the approval of the study protocol must also be provided in the manuscript.

Plants

Seed stocks	N/A.
Novel plant genotypes	N/A.
Authentication	N/A.

Flow Cytometry

Plots

Confirm that:

- [x] The axis labels state the marker and fluorochrome used (e.g. CD4-FITC).
- [x] The axis scales are clearly visible. Include numbers along axes only for bottom left plot of group (a 'group' is an analysis of identical markers).
- [x] All plots are contour plots with outliers or pseudocolor plots.
- [x] A numerical value for number of cells or percentage (with statistics) is provided.

Methodology

Sample preparation	For flow cytometry analysis of algae-PNP(Vanc)-robot, after platelet membrane coating onto the DiO-loaded nanoparticles, nanoparticles were conjugated to algae via click chemistry. Details on algae-PNP(Vanc)-robot preparation are provided in Methods section. For flow cytometry analysis of algae robot uptake by alveolar macrophage in vivo, bronchoalveolar lavage fluid was collected from each mouse. RBCs in the mixture were lysed before the cells were blocked with 1% bovine serum albumin and probed with Pacific Blue-conjugated anti-mouse CD11c and PE-conjugated anti-mouse Siglec-F antibodies. Unbound antibodies were washed out with 1% bovine serum albumin prior to analysis. More details are provided in Methods section.
Instrument	Becton Dickinson FACSCanto-II flow cytometer.

Software	FlowJo 7.6
Cell population abundance	For algae robot analysis, 100,000 total events were acquired and for alveolar macrophage analysis, 20,000 cells were collected.
Gating strategy	For algae-PNP(Vanc)-robot characterization, preliminary FSC/SSC gates were determined by comparing the population of control sample (unmodified algae robot) with positive algae-PNP(Vanc)-robot sample. For algae robot uptake by alveolar macrophage in vivo, cellular debris were gated out on FSC/SSC plots and doublet exclusion was performed. Specific positive populations were identified by comparing the population with single stained control samples (i.e. cells labeled with only one antibody or algae only).

☒ Tick this box to confirm that a figure exemplifying the gating strategy is provided in the Supplementary Information.
